# Effects of Consensus on Leader–Member Exchange (LMX) Within Nursing Teams on the Relationship Between Abusive Supervision, Job Satisfaction, and Unit Turnover: A Multilevel Moderation Study

**DOI:** 10.1155/jonm/6220416

**Published:** 2025-04-01

**Authors:** Andrea Caputo, Patricia Costa, Paola Gatti, Claudio G. Cortese, Aristides I. Ferreira

**Affiliations:** ^1^Psychology Department, University of Turin, Turin 10124, Italy; ^2^Business Research Unit (BRU-IUL), Iscte—Instituto Universitário de Lisboa, Lisbon 1649-026, Portugal; ^3^Psychology Department, University of Milano-Bicocca, Milan 20126, Italy

**Keywords:** abusive supervision, climate strength, consensus, job satisfaction, LMX, multilevel moderation, turnover intentions

## Abstract

**Aim:** This study explores how consensus on leader–member exchange (LMX)—the degree of within-unit agreement regarding the LMX nurse leaders establish with each team member—moderates the effects of abusive supervision on job satisfaction and internal turnover intentions.

**Method:** Involving a sample of 1357 nurses nested into 130 groups (led by as many nurse leaders), cross-level moderations were tested.

**Results:** Results show that, on one hand, LMX consensus acts as a resource when it is stronger, dampening the effect of abusive supervision on job satisfaction. On the other hand, nurses with higher job satisfaction belonging to groups with higher LMX consensus report higher intentions to change wards than nurses in groups with lower LMX consensus. The discussion addresses the concept of “star employees,” i.e., employees with better performance, visibility, and relevant social capital.

**Implications for Nursing Management:** The discussion highlights the importance for nurse managers to consider both the quality of individual LMX and overall team consensus to enhance nurse well-being and reduce turnover intentions.

## 1. Introduction

While the beneficial effects of positive leadership behaviors in enhancing nurses' attitudes and behaviors, along with patient satisfaction and quality of care, are well-known [1], healthcare organizations and leaders continue to face issues related to nurse well-being and turnover. In particular, since nurse leaders play a key role in influencing nurses' well-being [2], it is vital for nurses' perceptions about the behaviors of their leaders to be addressed, with a view to protecting their well-being. This is also a key feature in which to invest to improve nurse retention [3], as abusive supervision is one of the main antecedents prompting nurses to leave [4]. According to the literature [3], the increasing turnover rate of nurses is a concerning issue. In Italy, for example, there was a progressively higher turnover rate between 2010 and 2019, with exiting nurses not being replaced in sufficient numbers1. This trend worsened in the post-COVID-19 period, with a significant percentage of nurses leaving the National Health Service (NHS). Specifically, 56% of Italian nurses reported considering leaving the NHS, citing factors such as burnout, unsustainable workloads, and dissatisfaction with working conditions2. In addition to the situation in Italy, nurse turnover remains a significant challenge globally, with considerable variation across regions. In North America, for instance, the turnover rate among registered nurses was 18.4% in 2023, showing a slight decrease from the previous year. Certain specialties, such as emergency and telemetry, have experienced cumulative turnover rates as high as 119% over the past 5 years3. Similarly, in Asia, the turnover rate among nurses in China is currently 15.6%, highlighting persistent challenges in retaining healthcare staff [4]. Thus, nurse turnover confirms to be a global issue, but it is also necessary to emphasize the need for region-specific strategies to address it.

Nurses' turnover intentions originate from within the work unit [5], and if nurses do not abandon the organization due to high demands and burnout, they may opt to leave their ward, seeking a different unit in which to work. However, such choices come with associated costs; when nurses leave their work unit, this leads to a need to readjust the workforce, thereby having implications on perceptions of stability and cohesion within the team, which, in turn, affects the quality of care [6].

Organizational literature has acknowledged the importance of nurse leaders on nurses' psychological and behavioral outcomes. For example, studies on the quality of the relationship between leader–nurse dyads (i.e., leader–member exchange; from now on we will use the acronym LMX) [7] have confirmed that high-quality relationships can reduce nurses' turnover intentions [8]. This relationship is not entirely detached from the other dyadic relationships established by nurse leaders with other nurses in the same work unit [9], since the evolution of the LMX theory moved beyond the concept of the “vertical dyad linkage” approach, which focuses only on the study of dyads within units, and conceives the group as an aggregation of dyads [7]. Therefore, well-being and behavioral outcomes can be affected not only by the quality of the relationship itself but also by similar perceptions among other nurses about the leader establishing relationships of different (or the same) quality within the group. In this area of research, our study aims to investigate how nurses' consensus on the quality of the relationship with the same leader in their working unit affects their work-related satisfaction and any intentions they may have to leave the ward.

The act of quitting the organization is the culmination of a process in which “the work group can be considered as the first (work) place environment where employees develop cognition of leaving” [5, p. 755]. Since organizations are made up of a series of work groups, it would be restrictive not to consider the influence of group-level dynamics, mainly within a sample of nurses who develop strong relationships with their colleagues and supervisors on a daily basis [5]. Thus, exploring the moderating effects of LMX, conceived as group-level leadership climate strength [10], could both extend the climate strength concept to other constructs [11] and add new nuances to our understanding of individual-level leadership dynamics influenced by group-level consensus [12].

### 1.1. The Effects of Abusive Supervision on Job Satisfaction and Internal Turnover Intentions

Abusive supervision is defined as “subordinates' perceptions of the extent to which supervisors engage in the sustained display of hostile verbal and nonverbal behaviors, excluding physical contact” [13, p. 178]. According to this negative leadership style, leaders are perceived as tyrannical bosses who undermine and underestimate their followers. Indeed, the behaviors linked to this style include, among others, public criticism, rudeness, and coercion. Nurse leaders who exhibit abusive supervision may demonstrate indifference toward their followers, engage in rude or dismissive communication, raise their voices while talking to nurses, publicly belittle their nurses to undermine their self-esteem, or even employ the silent treatment. These behaviors not only harm the psychological well-being of nurses but also create a toxic work environment that can lead to reduced job satisfaction and increased turnover intentions [14].

Studies focusing on abusive supervision, specifically encompassing personal attacks, consistently indicate that these attacks not only contribute to weakening job satisfaction but also result in increased levels of psychological strain and intentions to quit [14]. Interest in research about the features of the dark side of leadership is linked to the costs for organizations associated with destructive leaders' behaviors, considering both the economic consequences—connected to decreased productivity, greater absenteeism, and higher healthcare costs—and the psychological outcomes for employees [15]. Some negative effects associated with these abusive types of leadership behaviors include emotional exhaustion [16] and increased intention to quit [13].

In narrowing the focus to our study's variables of interest, the literature has highlighted the negative effects of abusive supervision on job satisfaction, which also leads to turnover intentions [17]. Looking more specifically at healthcare workers, nurses frequently experience the negative consequences of abusive supervision in weakening their own job satisfaction, as demonstrated by research in various national contexts [18]. At the same time, turnover intentions and turnover rates, both outside and inside the organization, have been a major issue in nursing workforce management for many years [3].

In the nursing literature, job satisfaction has often been studied as the mediator of the relationship between abusive supervision and some psychological and organizational outcomes, such as nursing quality of care [18] and intentions to quit [14]. Job satisfaction is defined as one's attitude or affect toward a job, and, if negatively affected, it is a crucial construct for explaining why nurses' turnover intentions tend to increase [19]. The mediating role of job satisfaction has been widely explored, mainly considering the nursing workforce [20]. Numerous studies in various national contexts have explored how job satisfaction can mediate the relationship between different kinds of antecedents, all influencing one of the most explored outcomes in nursing studies, i.e., turnover intentions [21]. For example, Lu and colleagues [20] offer a review of the different antecedents and outcomes of job satisfaction among hospital nurses, also highlighting its mediating role. Other studies have also explored the effects of LMX on job satisfaction, which mediates the relationship between LMX and turnover intentions [8].

Widely recognized as one of the main issues in healthcare contexts, turnover is defined as the process whereby nurses leave the organization (external turnover) or transfer within their own work organization (internal turnover), thus changing either organization or ward [3]. Turnover intentions arise as psychological, cognitive, and behavioral responses to negative conditions experienced by employees, due to which they seek new and improved conditions outside the organization, by finding a new job or by changing working unit. In Italy, for example, nurses tend to abandon the public sector to seek private employment, due to the critical conditions of high job demands and burnout4. The negative antecedents of this kind of organizational outcome include abusive supervision [22]. There are various explanations about what leads to the increase in nurses' turnover intentions when faced with an abusive leader: for example, a recent study highlighted the mediating role of diminished leadership, organizational identification, and job satisfaction itself [14].

Leadership definitely plays an important role in affecting turnover intentions; in fact, previous studies have highlighted that LMX within the unit, i.e., the quality of interaction in the nurse–supervisor relationship within the specific work unit, significantly affects internal turnover among nurses [23]. When considering the relationship between nurses within the individual working unit, and thus with their direct manager, it can be seen that unit-level nursing supervisors play a crucial role in providing work environments that promote nurses' well-being, influence the quality of support within the nursing unit, and thus ensure high-quality patient care [24]. Nevertheless, although prior research has looked into the impacts of LMX measured at the unit level, our study will focus in this area on a particular unit-level LMX, albeit examining a distinct indicator closely associated with the climate strength literature. It is not only the quality of LMX at the unit level that affects organizational outcomes and well-being, but also the degree of within-group consensus among team members regarding perceptions of LMX quality. As suggested by the literature on climate strength, this consensus can influence those outcomes.

Alongside the turnover phenomenon whereby employees leave the organization, internal turnover can also be a crucial issue for organizations, particularly those in the healthcare sector. Internal turnover is a concept linked to job turnover, also labeled “unit turnover” [25]. It refers to employees leaving their current role, moving to new positions or taking on different responsibilities within the organization. Thus, the concept of unit turnover refers to individuals working in the same role but in a different work unit within the same organization such that the terms “unit turnover,” “internal turnover,” or “unit-level turnover” can be used interchangeably [25]. In this study, internal turnover intentions refer to nurses' intention to change to a different department or unit in the organization, keeping the same job title or role.

Within the healthcare context, nurses' turnover is a critical issue [6]. According to turnover theory, the process of intending to leave the organization may start with contemplating the idea of exiting the immediate work group. Subsequently, this intention may extend and evolve into a broader decision to leave the organization as a whole [23]. Hence, the work unit is a critical context that influences major decisions on a larger scale. A nursing unit is characterized by the interactive context shaped by nurses' attributes, exchanges, and responses [6]. Within this framework, nurses establish working relationships with their peers, supervisors, and also physicians. Therefore, the quality of these relationships can exert a substantial influence on nurses' attitudes and decisions about their careers [23]. Accordingly, and in line with social exchange theory [26], the relationship established by nurses within their working unit with physicians or with their direct supervisors is the antecedent of their health, satisfaction, productivity, and retention. Indeed, nurses who have established a good dyadic relationship with their supervisor contribute to improving retention indices [23]. On the other hand, nurses' internal turnover significantly affects patient outcomes, such as patient satisfaction or medication errors. Moreover, nurses' unit turnover poses a challenge as it involves organizational costs and requires subsequent readjustments, including recruiting new staff and addressing the perceptions of weakened workgroup cohesion among the remaining nurses [6].

The relationship between abusive supervision, job satisfaction, and internal turnover intention can be further understood through the framework of the Conservation of Resources (COR) theory [27]. According to the COR theory, individuals strive to acquire, retain, and protect valued resources. Stress arises when individuals perceive a threat to their resources or experience actual resource loss. As a coping strategy, individuals may either confront the situation or withdraw from it to preserve their remaining resources [28]. In a managerial context, a leader exhibiting abusive behaviors may threaten these resources through hostile actions, increasing employees' stress levels, decreasing their job satisfaction, and subsequently affecting their work and productivity [29]. This, in turn, may prompt employees to adopt behaviors aimed at conserving their resources, such as increasing their turnover intentions [28].

Thus, having explored the negative effects of abusive supervision on job satisfaction, its increasing effects on turnover intentions, and the mediating role of job satisfaction between leadership constructs and nurses' intentions to leave the organization, we ultimately hypothesize that

• H1: Abusive supervision has an indirect positive effect on internal turnover intentions via job satisfaction at the within level.

### 1.2. Climate Strength and LMX Within-Unit Consensus

Organizational climate refers to the collective perceptions and significance attributed to policies, practices, and procedures encountered by employees at work, along with the behaviors that receive recognition and are expected and endorsed [30]. Typically, when studying organizational climates, researchers use aggregated means of measuring individual employees' dimensions analyzed at the unit level, since each unit within an organization has its own climate, i.e., agreement or consensus in perceptions, that differs from other units' climates [31]. According to Chan's [32] models, this refers to the *direct consensus* model, in which within-group consensus of the lower-level units is considered “the functional relationship to specify how the construct conceptualized and operationalized at the lower level is functionally isomorphic to another form of the construct at the higher level” (p. 237). In studies using this approach, within-group agreement or consensus serves to justify the aggregation of scores at the lower level to represent scores at the higher level [33].

However, Chan [32] also proposes a more interesting model according to which within-unit agreement is used as the main construct to analyze, i.e., the *dispersion model*. In this definition, dispersion (and within-unit agreement or consensus, which is its opposite) refers by definition to a unit-level construct, operationalized in this logic, since it represents the variability within a group. For explanatory purposes, the group-level construct to be analyzed is the variance of the individual-level variables [34]. Thus, while direct consensus models measure the level of a construct, dispersion models measure the extent to which the construct varies within teams [35]. Methodologically speaking, in the dispersion model, within-unit variance “is treated as a meaningful higher-level construct rather than a statistical prerequisite for aggregation” [36, p. 384]. Furthermore, the dispersion model relies on the dispersion theory put forward by Brown and Kozlowski [37], according to which social interaction processes allow individual-level constructs to emerge as unit-level constructs, such that within-unit consensus is considered the degree to which unit-level constructs arise [33]. For these reasons, research into organizational climates tends to use another index, which follows the dispersion model, such as *climate strength*.

Climate strength is defined as “the degree of within-unit agreement among unit members' climate perceptions” [33, p. 465]. This construct allows researchers to answer questions about the implications of variability in consensus within teams [30], since it determines the degree of consensus between individuals' perceptions of climate within the same team. For example, stronger consensus on innovation climate intensifies work satisfaction [33] and weaker consensus on procedural justice climate intensifies team absenteeism [38]. Climate strength is linked to the situational strength concept [34], according to which a distinction is made between strong and weak situations. In the literature, researchers refer to “strong situations” when people in the same team or unit perceive events in the same way; therefore, there is high consensus within the unit and low variability, i.e., high climate strength. Conversely, they refer to “weak situations” when team members are characterized by a high degree of variability in their perceptions [9]. According to studies into the Human Resource Management System (HRMS), strong climates arise when management is able to lead people strongly, while weak climates are created when there is weak consensus among employees about, e.g., the organizational vision [35, 39].

The literature on climate strength has demonstrated its moderating role between climate and some organizational outcomes, using different constructs operationalized as climate strength. For example, the moderating effect of innovation climate strength on the relationship between innovation climate and organizational commitment and work satisfaction [33] has been demonstrated. Other studies have shown that high climate strength (i.e., high consensus) among nurses better predicts safety outcomes [40]. Usually, these studies, where climate strength moderates the relationship between climate and outcomes of interest, show that the relationship is stronger when climate strength is high, i.e., strong situations [30].

Beyond exploring antecedents of climate strength, some studies have focused on climate strength operationalizations with leadership constructs, exploring the effects of variability in the followers' perceptions of their leaders' behaviors [41]. In these cases, the climate strength literature has highlighted the importance of having shared perceptions between team members. Some authors working on climate strength have highlighted the closeness of leadership and climate constructs, sometimes referring to these concepts as “leadership climate” [cf. [42]]. However, Schneider and colleagues [11] have perhaps demonstrated that this is, in fact, an inappropriate term to use. Therefore, we explore the effects of leadership consensus [43].

*Leadership consensus* is defined as “the shared perceptions of employees toward their direct supervisor” [12, p. 104]. This concept was studied to capture team-level consensus on some leadership constructs, such as leaders' expression of humility [41] and transformational leadership [43]. While expressing perceptions about leadership, followers could report high or low levels of, for example, transformational leadership or LMX, but at the same time, they could express a high (or low) level of climate strength since they may or may not share common perceptions about their leader's leadership style or the relationship quality [43]. When consensus is high, team members perceive leaders' behaviors or attitudes homogeneously; on the contrary, when consensus is low, there are dissimilarities in followers' perceptions of leaders' behaviors or attitudes [36]. For example, stronger consensus on the leader's transformational leadership enhances the leader's role in developing team cohesion in a sporting environment [43] and empowerment in the organizational context [36].

### 1.3. The Moderating Role of LMX Consensus Among Followers

Leadership literature has strongly acknowledged the importance of leaders in shaping followers' psychological outcomes [2] and in influencing followers' team climate perceptions [33]. Leadership is substantiated in the relationship with followers [44]; a further measurement of leadership effectiveness could be the degree of consensus created between followers about leaders' behaviors, i.e., facilitating similar perceptions between team members [36]. Therefore, according to Chan's [32] dispersion model, the degree of consensus among followers of the same working team about the quality of the relationship with their leader could affect followers' psychological constructs and organizational outcomes of interest for firms [36]. According to LMX theory [7], leaders establish different relationships with followers, acting differently according to the follower with whom they are interacting. Since leaders, during their relationship with followers, develop qualitatively different interactions, LMX has been analyzed by studying leader–follower dyads [10]. High-quality leader–member relationships are characterized by leaders providing their followers with increased resources, confidence, support, and autonomy. Some meta-analytic studies on LMX have demonstrated that high-quality relationships at the individual level can facilitate positive work outcomes, including enhanced performance, job satisfaction, organizational commitment, and expression of organizational citizenship [45].

However, LMX should not be studied as a dyadic phenomenon alone, since in working groups, leader–follower relationships could be considered “as a system of interdependent or interwoven dyadic relationships in work networks, rather than as a set of independent dyads” [9, p. 2]. Therefore, since leaders establish different qualitative relationships with each follower, by definition of LMX, considering the within-group level means that there could be different levels of consensus between followers about LMX with their leader: Followers may believe that they are treated in the same way (high consensus) or in different ways (low consensus) by the same leader, and this has implications, for example, on perceived justice or fairness [10]. In fact, low consensus on LMX among team-level followers creates within-group disagreement, whereby followers perceive that leaders are behaving, interacting, and communicating in different ways with each follower, and climate strength literature suggests that this disagreement negatively affects justice climate perceptions [39].

**Figure 1:**
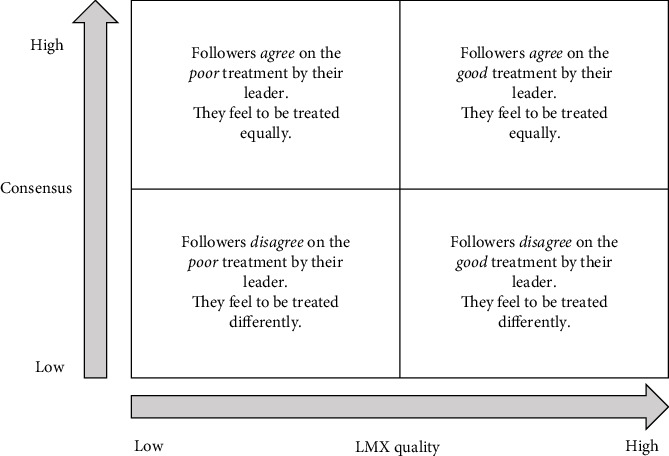
Perceptions of treatment under different conditions of consensus and LMX quality.

Therefore, while studying within-unit consensus, the focus is placed on followers, managed by the same leader, assessing the level of consensus (or lack thereof) on the quality of the relationship established with their leader, which could be high or low. There could, for example, be a high consensus on a low-quality LMX; in this case, followers share the same perceptions about their leader. On the other hand, when followers do not agree (low consensus, high variability), they may feel they are being treated in different ways, and this could affect different psychological and organizational outcomes [10].  Figure 1 clarifies the perceptions of satisfaction when high and low consensus and high and low LMX quality intersect.

Given the impact of within-group agreement and unit turnover, it is interesting to shift the research focus toward exploring the effects of within-unit consensus in leadership dynamics on individual well-being outcomes and individual intentions to move between units in the same organization. For this reason, we hypothesize that

• H2a: LMX consensus at the unit-level moderates the individual-level relationships between abusive supervision and job satisfaction such that abusive supervision has a weaker negative effect on job satisfaction within teams characterized by high (versus low) LMX consensus.
• H2b: LMX consensus at the unit-level moderates the individual-level relationships between job satisfaction and internal turnover intentions such that job satisfaction has a stronger negative effect on internal turnover intentions within teams characterized by high (versus low) LMX consensus.
• H2c: LMX consensus at the unit-level moderates the individual-level relationships between abusive supervision and internal turnover intentions such that abusive supervision has a weaker positive effect on internal turnover intentions within teams characterized by high (versus low) LMX consensus.

**Figure 2:**
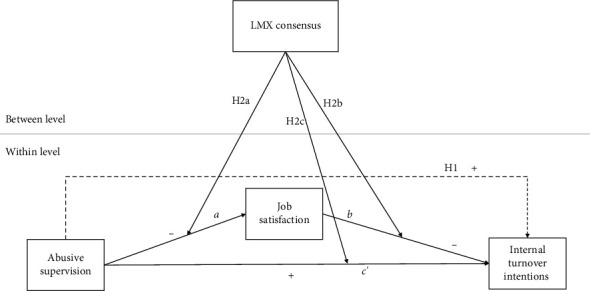
The hypothesized model.

Figure 2 shows the hypothesized model.

## 2. Method

### 2.1. Study Design

A sample of 1357 Italian nurses and 130 nurse leaders working in hospitals in northwestern Italy was involved, by filling out paper-and-pencil questionnaires. This study is part of a broader project entitled “Feeling like a leader” whose focus was the study of leadership relationship features between nurse leaders and nurses of their working groups. To match and merge the data of nurse leaders with their respective followers' groups and at the same time respect the confidentiality of participants' personal data, researchers generated alphanumeric codes. Participants were informed about the process through invitation letters and information sheets accompanying each questionnaire. Data collection started after the project obtained approval from the director of the Directorate of Health Professions and the nurse leaders of the target organization, allowing the participation of both leaders and their followers. Additionally, approval was granted by the Bio-Ethics Committee of the University of Turin (Approval letter, Prot. No. 55631, dated 1 February 2019). Nurse leaders were invited via email, accompanied by an information sheet introducing the research characteristics. Upon agreement to participate, two administrators delivered paper copies of the questionnaires in person, collecting them soon after the completion. Nurses filled out their questionnaires, signed them with an alphanumeric code, and placed them in blank envelopes. The administrators collected all the envelopes from each ward. The study findings were reported in compliance with the Strengthening the Reporting of Observational Studies in Epidemiology (STROBE) statement for cross-sectional studies [46].

### 2.2. Measures

*Abusive supervision* was measured using the Italian validated version of Tepper's [13] 15-item scale [47], with a 5-point Likert scale, ranging from 1 (“*I cannot remember him/her ever using this behavior with me*”) to 5 (“*He/she uses this behavior very often with me*”). A sample item is “My nurse leader gives me the silent treatment,” McDonald's *ω* = 0.92.

*Job satisfaction* was measured with a 5-item scale, 4 items from the Copenhagen Psychosocial Questionnaire (COPSOQ) [48]. Items were adapted to the Italian language and used in previously published studies [19]. An example item is “How satisfied are you with the way your abilities are used?”, and one ad hoc item asks “How satisfied are you with your work as a whole, taking into consideration each element?”. Respondents were asked to answer using a 5-point Likert response scale, from 1 (“*Very dissatisfied*”) to 5 (“*Very satisfied*”). McDonald's *ω* = 0.87.

*Internal turnover intention* was measured by adapting the formulation of the 3-item intention to quit and turnover scale by Colarelli [49] with statements regarding changing the ward within the same organization. Participants were asked to answer using a 5-point Likert scale from 1 (“*Strongly disagree*”) to 5 (“*Strongly agree*”). An example item is “If I have my own way, I will be working for another unit/ward within this organization a year from now,” McDonald's *ω* = 0.91.

*LMX* was assessed with the 7-item scale by Graen and Uhl-Bien [7], with a 5-point Likert response scale, which statement changes according to the item. The items were adapted to Italian. An example item is “Do you usually know how satisfied your leader is with what you do?”, McDonald's *ω* = 0.92.

Then, in order to measure *LMX consensus* for every ward, the mean Euclidean distance was used, calculating the index with the following formula: ∑sqrt[∑(*Si* − *Sj*)^2/*n*]/*n*. With this index, we averaged the dyadic differences between each individual participant (*Si*) and the other members within the same working group (*Sj*), then we aggregated all the scores for dissimilarities for each ward ranged from 0 to 0.99, and finally, reversing the score, we show that higher scores mean greater consensus. This approach is grounded in Chan's [32] dispersion model, which conceptualizes consensus as a team-level configurational property [50]. The use of the Euclidean distance specifically allows us to capture the variability in members' perceptions by measuring the separation between individuals' views and those of their group members. As highlighted by Harrison and Klein [51], this method provides a robust representation of lateral differences within a unit, reflecting the root mean squared distance across all dyads. By operationalizing consensus in this way, we emphasize dissimilarity within the group, enabling a more accurate assessment of alignment in LMX perceptions among team members.

### 2.3. Data Analysis

Data analysis was performed using SPSS 28 (IBM, Armonk, NY, USA) for descriptive statistics, reliability and correlational analysis, the Excel tool from Biemann and colleagues [52] for r_WG(J),_ ICC_(1)_, ICC_(2)_, and D_eff_, and MPlus 8 (Muthén and Muthén, Los Angeles, CA, USA) for multilevel analyses.

“Leadership is inherently multilevel in nature” [53, p. 4], as individuals within a work group led by the same leader are “nested” within that leader. In multilevel studies, Level 1 (individual level) refers to individual perceptions and outcomes, while Level 2 (group level) comprises aggregated group-level variables, derived from individual responses within the same work unit. Leadership, as a social construct, operates across individual and group contexts. Leaders interact with individuals (Level 1) while simultaneously influencing the broader team environment (Level 2), and the multilevel model enables partitioning of variance between levels, ensuring robust estimates.

To test the multilevel moderated mediation model, with the moderator (i.e., LMX consensus) at the between level and all the variables implied in the mediation model, firstly, we group mean-centered the Level 1 predictor (i.e., abusive supervision) and grand mean-centered the Level 2 predictor (i.e., LMX consensus), and then, we used the “two-level random” type of analysis in Mplus, specifying “algorithm = integration” [54, 55]. The two-level random approach in multilevel moderated mediation allows the random slope at Level 1 to be considered a latent variable at Level 2, used as an outcome variable to test the interaction effect [56]. This approach aligns with recommendations from the psychometric literature on analyzing multilevel models where both intercepts and slopes are considered outcome variables in the cross-level moderation model [57]. To test the moderated mediation hypotheses of abusive supervision on internal turnover intentions via job satisfaction, with different conditions of LMX consensus moderating the different paths (a, b, and c'), i.e., high and low LMX consensus, we used the bootstrap estimates with the “integration = montecarlo” input to construct bias-corrected confidence interval (CI).

In order to justify aggregation and the multilevel analyses, r_WG(J),_ ICC_(1)_, ICC_(2)_, and *D*_eff_ were calculated [53, 58]. r_WG(J)_ is the index of within-group interrater agreement, which assesses “agreement among the judgments made by a single group of judges on a single variable in regard to a single target” [59, p.5; 60]; ICC_(1)_ expresses the portion of the total variance of the variables that significantly vary across teams, i.e., localized in the upper level; besides it, ICC_(2)_ indicates the reliability of the scores at the higher-level analysis; finally, the design effect (*D*_eff_) measures how much the observations deviate from a hypothetical simple random sampling [61]. We adopted the following cutoff values: r_WG(J)_ between 0.51 and 0.70 shows a moderated agreement, while values greater than 0.70 show consistent agreement [58]; for ICC_(1)_, values greater than 0.05 are considered acceptable, while for ICC_(2)_, they should be greater than 0.70 [53]; finally, values of *D*_eff_ greater than 2 suggest the non-negligibility of clustering effects on the variables [61]. Our study showed sufficient values to proceed to multilevel analysis. Abusive supervision showed an r_WG(J)_ of 0.91; ICC_(1)_ was 0.08 and ICC_(2)_ was 0.52. For job satisfaction, r_WG(J)_ was 0.82, ICC_(1)_ was equal to 0.08, and ICC_(2)_ was equal to 0.55. Then, internal turnover intention showed an r_WG(J)_ of 0.33, an ICC_(1)_ of 0.11, and an ICC_(2)_ of 0.61. Lastly, LMX showed an r_WG(J)_ equal to 0.85, ICC_(1)_ was 0.19, and ICC_(2)_ was 0.70.

## 3. Results

### 3.1. Characteristics of the Study Participants

The whole population of nurse leaders and nurses of the organization target was involved, counting 164 nurse leaders and 2664 nurses. Inclusion criteria in this study's sample were nurse leaders consenting to complete the questionnaires, a minimum of three nurses per group participating in the questionnaire, and ensuring that at least 61% of all items were completed comprehensively. According to these criteria and after cleaning the data matrix from missing data, the final sample counts 1357 nurses nested into 130 groups (led by as many nurse leaders). The sample of nurses is comprised of 82.2% women and 17.8% men; the age range has a minimum of 22 and a maximum of 66 years (mean = 43.52, SD = 8.98); 53.6% have a nursing school diploma, the 41.9% a bachelor's degree, and a 4.6% a master's degree. Nurses in the sample work within a university hospital network consisting of four large hospitals. Specifically, 51.6% of respondents are employed in the largest hospital district, and the remaining 20.5% in the trauma center hospital, 16.7% in the pediatric hospital, and 7.4% in the obstetrics and gynecology hospital. Additionally, 3.8% work in other hospitals located near the urban area. Moreover, 64.3% work in the general medicine clinical area, while 28.4% in surgery, 14.4% in emergency room, and 19.9% in pediatrics. As regards specific wards, 22.9% work in general and specialized surgery, 21.4% in oncology, 11.9% in gynecology and obstetrics, 11.5% in pediatrics, 10.7% in orthopedics and rehabilitation, 9.2% in neurosciences, 5.8% in general and specialized medicine, 2.9% in emergency and intensive care, and 3.7% in other wards. Nurses have been working in the organization for an average of 17.57 years (SD = 9.74), while they have been working for their specific ward for a minimum of 1 year to a maximum of 41 years (mean = 10.71, SD = 8.48). Briefly, nurse leaders are composed of 84.6% women and 15.4% men, with an average age of 53.1 years (SD = 5.4). Among them, 58.5% work in the largest hospital district, 16.9% in the trauma center hospital, 16.2% in the pediatric hospital, 6.2% in the obstetrics and gynecology hospital, and 2.3% in other hospitals near the urban area. On average, nurse leaders have been coordinating their ward for 13.1 years (SD = 8.4) and have a total work experience of 32.6 years (SD = 6.27).

As regards the representativeness of the sample, its characteristics align with the general profile of nurses in Italy. Specifically, the gender distribution reflects the predominance of women in the nursing profession, at both the national (76.4%) and the specific regional levels (84.4%)5. Similarly, the age distribution corresponds to the national trend of an aging nursing workforce, with a significant proportion being in their 40s or older (mean = 56.5 years old)6.

### 3.2. Results of the Model Analyses

Table 1 summarizes the descriptive statistics and the correlations of the study variables.

Descriptive analyses highlight some notable findings worth discussing. For instance, the low r_WG(J)_ value for internal turnover intentions suggests substantial individual-level variance within groups. This aligns with the theoretical understanding that turnover intentions are inherently subjective and driven by personal circumstances, preferences, and experiences. Unlike variables such as job satisfaction or LMX, which may be shaped by shared perceptions of the work environment or leadership style, turnover intentions are more influenced by individual-level psychological and career-related considerations. This variability within groups likely reflects the heterogeneity in how individuals interpret shared experiences or weigh personal factors (e.g., career aspirations, family obligations) when deciding whether to stay or leave.

Regarding ICC_(2)_ values, while some fall below the conventional 0.70 threshold, we justified the aggregation of group-level variables by combining ICC_(1)_, r_WG(J)_, and theoretical considerations. The ICC_(1)_ values indicate meaningful between-group variance, supporting the multilevel approach. ICC_(2)_ values below 0.70 indicate that the aggregated group-level scores may have moderate reliability. In our study, lower ICC_(2)_ values may reflect substantial within-group variability, particularly for constructs like internal turnover intention. However, we analyzed abusive supervision, job satisfaction, and internal turnover intentions at the within level, while employing LMX consensus—which met all thresholds for aggregation—as the sole group-level variable in a cross-level model. This approach ensures that individual- and group-level influences are appropriately modeled and interpreted. Lastly, *D*_eff_ values indicate how much the data's structure, such as clustering within groups, affects the standard errors of estimates compared to a simple random sample. *D*_eff_ values greater than 2 (such as those of LMX consensus) suggest that the intragroup correlation (variance within groups) is substantial, justifying the need for multilevel modeling.

Prior to testing our hypothesized model, confirmatory factor analyses (CFAs) were performed in order to ensure the independence of the variables and to check common-method bias through Harman's single factor method [62, 63]. For CFAs, the following cutoffs of the fit indices were followed: values of Comparative Fit Index (CFI) and Tucker–Lewis Index (TLI) > 0.90/0.95 indicate a good fit of the model; values of root mean square error of approximation (RMSEA) < 0.05/.08 indicate an acceptable fit (while scores < 0.10 indicate a mediocre one) and of standardized root mean square residual (SRMR) < 0.08 [64]. A model in which variables load into only one factor shows not-so-good fit indices (*χ*_(435)_^2^ = 14,125.22, *p* < 0.001; CFI = 0.49; TLI = 0.45; RMSEA = 0.11 [0.111; 0.115]; SRMR = 0.13), while the model considering four different dimensions shows better fit (*χ*_(435)_^2^ = 14,125.22, *p* < 0.001; CFI = 0.90; TLI = 0.89; RMSEA = 0.05 [0.048; 0.053]; SRMR = 0.21), except for SRMR values. Therefore, considering the multilevel nature of our model, we performed a multilevel CFA, where only LMX consensus is analyzed at between level. Multilevel CFA shows a better fit (*χ*_(253)_^2^ = 7680.60, *p* < 0.001; CFI = 0.92; TLI = 0.91; RMSEA = 0.05; SRMR_whithn_ = 0.05; SRMR_between_ = 0.00). Then, we proceeded testing the hypothesized multilevel moderated mediation model. The results are shown in Table 2.

Results show that at the individual level, abusive supervision negatively affects job satisfaction and it is positively related to internal turnover intentions; in turn, job satisfaction is negatively related to turnover intentions. Finally, the indirect effect of abusive supervision on internal turnover intentions via job satisfaction at within level is positive and significant (unstandardized estimate = 0.18; *p* < 0.001). Thus, Hypothesis 1, at the individual level, is confirmed. Then, adding the between-level LMX consensus index as moderator of the three paths (a, b, and c'), the results show that LMX consensus significantly moderates the relationship between abusive supervision and job satisfaction (Path a, confirming Hypothesis 2a), and the one between job satisfaction and internal turnover intentions (Path b, partially confirming Hypothesis 2b, since the moderation is significant but in the opposite direction than expected), while the moderation of the direct relationship between abusive supervision and internal turnover intentions is not significant (Path c', disconfirming Hypothesis 2c).

Since significant prediction of the moderation of Path a and/or b by a moderator on the between level represents a case of moderated mediation [65], we also tested the indirect effects of the abusive supervision (Level-1 predictor) on internal turnover intentions (Level-1 outcome) via job satisfaction (Level-1 mediator) considering different levels (i.e., −1 SD and +1 SD) of LMX consensus (Level-2 moderator), significantly moderating Paths a and b.

**Figure 3:**
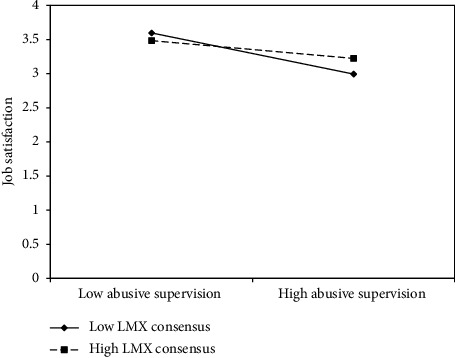
LMX consensus (at between level) moderating the relationship between abusive supervision and job satisfaction (at within level).

**Figure 4:**
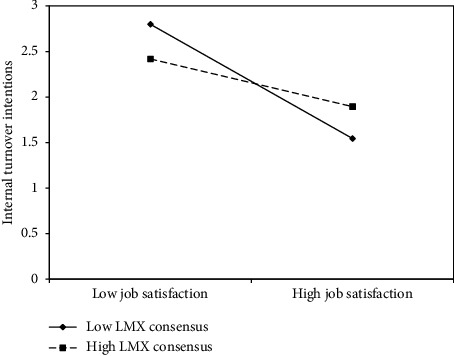
LMX consensus (at between level) moderating the relationship between job satisfaction and internal turnover intentions (at within level).

Firstly, we provide the visualization of the two significant moderations in Figures 3 and 4, i.e., the interaction plot of simple slopes.

Figure 3 shows the moderation of LMX consensus on the relationship between abusive supervision and job satisfaction, according to which this negative relationship was stronger for teams with lower LMX consensus than for teams with higher consensus, with groups characterized by lower consensus reporting lower levels of job satisfaction when abusive supervision increases, thus confirming Hypothesis 2a.

Furthermore, the indirect effect of abusive supervision on internal turnover intentions via job satisfaction, with LMX consensus moderating Path a, was calculated using the standard Monte Carlo replications to build 95% CIs. The results show that it is stronger for groups with low (−1 SD) LMX consensus (unstandardized estimate = 0.27; 95% CI [0.20; 0.27]) than for groups with high (+1 SD) LMX consensus (unstandardized estimate = 0.15; 95% CI [0.09; 0.15]).

Figure 4, instead, shows the LMX consensus role in moderating the relationship between job satisfaction and internal turnover intentions. In this case, nurses in teams with lower LMX consensus report lower intentions to change wards than nurses in teams with higher LMX consensus when job satisfaction is high. When consensus is low, the inverse relationship between job satisfaction and turnover intentions is stronger, thus partially confirming Hypothesis 2b. The moderating effect is, in fact, significant but in the opposite direction than expected, suggesting different effects of consensus on LMX between these two dimensions among a sample of nurses.

Furthermore, the indirect effect of abusive supervision on internal turnover intentions via job satisfaction, with LMX consensus moderating Path b, was calculated by bootstrapping 10,000 Monte Carlo replications to build 95% CIs. Results show that it is stronger for groups with low (−1 SD) LMX consensus (unstandardized estimate = 0.20; 95% CI [0.08; 0.31]) than for groups with high (+1 SD) LMX consensus (unstandardized estimate = 0.15; 95% CI [0.05; 0.26]).

## 4. Discussion

In confirming the well-known individual-level effects of abusive supervision in decreasing job satisfaction and increasing internal turnover intentions at the individual level [3], our study focuses on the role of consensus in LMX perceptions among teams in moderating the relationships between these dimensions. In line with our hypotheses, consensus acts as a resource when it is stronger, dampening the effect of abusive supervision on job satisfaction. Furthermore, the indirect effect of abusive supervision on internal turnover intentions via job satisfaction demonstrates that this relationship has a stronger influence within teams characterized by low consensus on LMX, suggesting that individuals tend to decide to change ward when they perceive they are being treated differently within their work units.

However, and more interestingly, LMX consensus has some unexpected effects in moderating the relationship between job satisfaction and internal turnover intentions. Our results show that nurses belonging to groups with a higher level of LMX consensus (i.e., where there are similar perceptions among team members about the quality of the relationship with the same leader) report higher intentions to change wards than those of nurses belonging to teams with less similar perceptions when job satisfaction is high. We expected consensus to have been a resource for nurses, increasing their willingness to remain within a group with high consensus, where there is agreement within the team. However, this unexpected result suggests a more interesting theoretical explanation for this attitude among nurses.

Lastly, our results show that consensus is not effective in moderating the relationship between abusive supervision and internal turnover intentions, suggesting that when negative leadership behaviors are implemented by the leader, it is largely irrelevant whether nurses within the same group are treated in the same way or differently, as they choose another group and another leader.

### 4.1. Theoretical Implications

This study offers theoretical contributions about the effects of abusive supervision on job satisfaction and internal turnover intentions, and about within-group consensus on individual indicators of nurses' well-being and organizational outcomes.

Firstly, although it is well-known that abusive supervision has a negative effect on job satisfaction and a reinforcing effect on turnover intentions [13], our study highlights that, at the individual level, abusive supervision also increases internal turnover intentions among nurses. Internal turnover in the healthcare sector presents dual challenges, involving psychological complexities associated with restoring team cohesion and economic consequences related to staff renewal [6, 23].

Secondly, our study expands the literature about the moderator role of consensus on a particular leadership dimension [66]. We explored the role of consensus among nurses in the same team about the quality of the relationship (LMX) established by each nurse with the same nurse leader. “LMX consensus” means that there are similar perceptions between nurses about the quality of the individual relationship established by each nurse within the same work group with the same leader, meaning that the nurses in that group perceive they are all being treated in the same way by the leader.

The fact of having shared (or not) perceptions about leadership within the team (a group-level variable) seems to have different effects on nurses at the individual level. It seems to act as a resource in the negative effects of abusive supervision on job satisfaction, dampening this effect for teams with high consensus; in this sense, the fact that nurses are part of a team that has similar perceptions about the quality of the relationship with the leader (whether negative or positive) seems to be a protection factor against experiencing abusive supervision, which could threaten nurses' job satisfaction. Indeed, previous studies have highlighted the role of consensus in strengthening team cohesion [43] and in maintaining higher levels of work commitment when emotional exhaustion increases [66].

Then, the analysis revealed that LMX consensus moderates the relationship between job satisfaction and internal turnover intentions, with a buffering effect. Specifically, when LMX consensus is high, the negative association between job satisfaction and internal turnover intentions is weaker. This suggests that consensus among team members regarding their relationship with the leader mitigates the extent to which job satisfaction influences nurses' desire to change wards. In contrast, when LMX consensus is low, the relationship between job satisfaction and internal turnover intentions is stronger, meaning that variations in job satisfaction have a greater effect on internal turnover intentions under conditions of disagreement about the leader's treatment of team members. The role of consensus as a resource seems to be present in the relationship between job satisfaction and internal turnover intentions, but only in the case of low job satisfaction. In this regard, it seems that disagreeing on the quality of the relationship with the nurse leader leads to higher internal turnover intentions when individuals' job satisfaction is low. When nurses have low job satisfaction and they also believe that the leader is treating each peer differently, this may be associated with low organizational justice [9], meaning nurses are more likely to change groups.

However, the role of consensus seems to change in conditions of high individual job satisfaction. In this case, when individuals live a satisfying job but the leader treats nurses in the same way (high LMX consensus), nurses may be more inclined to leave the group since, having reached a high level of satisfaction, they then need to strive further to reinforce their own well-being [67]. Even if LMX is related to justice perceptions [10], nurses reporting a higher level of job satisfaction do not seem to benefit from the leader treating all members equally, as the study shows that they report higher intentions to change ward, namely, to search for another team led by another leader, than nurses who perceive low LMX consensus. Why do satisfied nurses search for another team if they are already in a positive situation (i.e., satisfied with their job) within their work unit?

One explanation could lie in the concept of “star employees.” According to this idea, star employees are those workers within an organization “with disproportionately high and prolonged performance, visibility, and relevant social capital” [68, p. 624]. These characteristics are not seen “in absolute terms,” but “in relative terms,” which means that employees make differences between their peers and that these differences could make them feel like the star employee. That said, nurses who believe that they are all treated the same, without differentiation by the leader, may be motivated to search for a leader who is more able to recognize their individual efforts and merits. Recognition, indeed, could be the driver for star employees to search for another group. In line with self-determination theory [69], recognition is one of the aspects of employees' aspirations that can lead them to perceive more satisfaction at work, thus positively affecting their well-being. Furthermore, studies on healthcare providers show that recognition is one of the factors associated with job satisfaction [67], both influencing, in turn, nurses' turnover [3]. Consequently, reaching high levels of job satisfaction seems to be a starting point for nurses to seek motivational factors, as conceptualized by Herzberg's [70] motivational theory. According to this theory, hygiene factors relate to elements whose absence could lead to dissatisfaction (e.g., administration, salary, security, and working conditions), while motivational factors are related to internal motivation, which, like recognition, contributes to increasing employees' satisfaction [67, 68]. Star employees want to be recognized for their efforts, and once they have reached a certain level of job satisfaction, they are then more driven by elements linked to intrinsic motivation, such as recognition for their performance [67, 68]. Indeed, recognition is numbered among the motivational factors for the nurses' workforce [71]. Therefore, having high levels of job satisfaction and being treated just like the others may not be sufficient reasons to stay within the group. This is also consistent with optimal distinctiveness theory [72], which posits that individuals strive to simultaneously fulfill two fundamental needs: belongingness and uniqueness. On one hand, nurses desire to be treated by their leader in the same way that their leader treats other team members, thereby satisfying their need for interactional justice and belongingness [73]. On the other hand, those who perceive themselves as “star employees” seek to fulfill their need for uniqueness, wanting to be treated differently from others in recognition of their distinctiveness.

Thus, our research suggests different effects of consensus, considering relationships of different psychological dimensions and organizational outcomes. On the one hand, consensus about LMX seems to be a resource when faced with an abusive leader, capable of protecting nurses' job satisfaction; on the other hand, when experiencing high levels of satisfaction, the fact that everyone is treated the same does not appear to be a motivational lever for remaining within the same work unit for nurses. Indeed, driven by the motivation to stand out [68] and to be recognized [69], nurses may tend to search for another work unit, which is often the first step toward leaving the organization [5]. These results are important since turnover has been confirmed to be one of the major issues for healthcare organizations due to the COVID-19 pandemic [74]. Indeed, studies conducted on the consequences brought about by the pandemic have demonstrated that the lack of recognition by leaders was one of the main reasons linked to turnover intentions [75], suggesting both a deterioration of the problem and a greater need for leaders to pay attention to individual consideration and recognition of individual performances in order to strengthen nurses' retention.

### 4.2. Implications for Nursing Management

Within the healthcare context and the nurses' panorama characterized by high (internal and external) turnover, exploring the role of consensus on leadership characteristics and specifically on the quality of the relationship held by leaders with their followers may assist in gaining an understanding of how to address this problem affecting healthcare organizations. Interestingly, nurses compare their own relationship with the leader against that of other nurses. It is crucially important to pay attention to the relationship between nurses and nurse leaders within the specific working unit [23, 24]. It is even more important for leaders to be aware that these perceptions are related to perceived organizational justice and are linked to different well-being and organizational outcomes of interest. In employees' eyes, managers' behaviors are perceived as organizational practice, and perceiving to be treated inconsistently affects nurses' job satisfaction and organizational commitment [76]. On the one hand, it is positive for leaders to treat followers in the same way, in order to ensure that individuals perceive a climate of justice within the same teams; on the other hand, treating all followers in the same way may not always be positive, since there are also high-performance followers who, especially when they perceive high job satisfaction, could search for a leader who gives them more individual consideration and recognition, as they aim to stand out and be treated differently from the others, due to their skills. Recognizing star employees and treating them differently, in certain circumstances, could be powerful training for leaders to prevent internal turnover intentions.

Importantly, these implications are linked to the clinical needs of nurses, who face high workloads, stress, and organizational challenges daily. Improving nurse managers' leadership by reducing abusive supervision practices can have a tangible impact on lowering turnover intentions by fostering a work environment that prioritizes fairness, support, and recognition. For instance, tailored leadership approaches could address the diverse needs of nurses at different career stages. Novice nurses, who may be more vulnerable to burnout and uncertainty, require structured guidance, mentorship, and emotional support to facilitate their integration into the team and the profession. Conversely, experienced nurses may benefit from greater autonomy, recognition of their expertise, and opportunities to take on leadership roles within their units. Such differentiated management strategies could ensure that all nurses feel valued and engaged, thus ultimately enhancing retention.

These implications could help healthcare organizations gain a better understanding of the relationship between nurses and their nurse leaders and the different effects of LMX consensus in preventing internal turnover. Training programs for leaders should be designed to make them aware of the different behaviors that need to be implemented in order to encourage nurses' retention within the healthcare system, which is characterized by numerous obstacles (e.g., nursing shortage, high levels of turnover, and an aging nursing workforce) [77]. Leadership styles and behaviors are one of the antecedents linked to preventing turnover within the nurse leader–nurses relationship [77]. The use of leadership consensus variables could be useful for leaders in understanding the quality of the climate within their specific team and also when differentiating their relationship with specific followers to ensure they are more engaged and motivated to remain in the same working team, increasing retention indices for healthcare organizations.

### 4.3. Limitations and Future Studies

Some limitations have to be acknowledged. Firstly, the use of self-reporting measures raises a concern about common-method bias. For this reason, we provided the results of Harman's single factor test in the result paragraph. Secondly, this research employs a cross-sectional design, limiting the ability to draw conclusions about the causal relationships between the variables under investigation. Thirdly, the model lacks an evaluation of the effect of certain control variables at both levels, which may act as confounding factors. We tested the correlation between the study's key variables and several control variables, such as gender, age, tenure in the specific ward, tenure in the hospital, hours worked, and affiliation with hospital macroareas (Level 1), as well as the number of nurses in the team (Level 2). The Level 1 control variables either showed no correlation with the key study variables or only weak correlations (e.g., age and tenure in the hospital), without significant effects in the moderation model. Similarly, the Level 2 control variable had no significant effect in the moderation model. Therefore, we opted for a more parsimonious model, excluding the effects of control variables. Future studies could consider adding some of these control variables into similar models, potentially examining other variables not measured in our study, such as nurses' experience in nonpublic healthcare contexts or the number of nurse leaders they have worked with. Additionally, future studies could explore group perceptions of individual-level outcomes across various cultural contexts, i.e., in other countries, thereby addressing the influence of cultural differences. Fourth, the generalizability of our findings may be influenced by cultural differences in leadership perceptions and behaviors, as highlighted by cross-cultural research, such as the GLOBE project [78]; thus, future studies could replicate our research in different cultural and healthcare settings to explore potential variations in the observed effects of the moderating variable. Lastly, it was not possible for us to distinguish followers while highlighting star employees according to some performance indices.

Studies in the literature have highlighted the role of high-quality LMX relationships in enhancing job satisfaction and reducing turnover using a longitudinal approach [79]. Hence, following this example, future studies could investigate the effects of LMX consensus on job satisfaction and turnover, with a longitudinal design, which could provide valuable insights into the long-term effects of abusive supervision on turnover intentions and explore whether LMX consensus serves as a protective factor over time. Moreover, future research could analyze the differences between nurses perceiving themselves or being perceived either by their peers or by the leader as star employees according to some organizational outcomes of interest starting from the perception of well-being. Since star employees are those with the highest levels of performance, visibility, and relevant social capital [68], nurse leaders could identify nurses who excel in their work performance. This could be based on aspects such as patient satisfaction, possessed competencies, and social capital—conceptualized as the number and quality of interpersonal relationships the nurse establishes with colleagues and within the organization, i.e., how well-known and appreciated they are. This approach would allow distinguishing star employees from nonstar employees according to the perspective of the referring nurse leader [68]. Exploring the role of consensus on LMX and the differences between star employees and other workers could be useful for testing how the effect of consensus about the relationship with the same leader varies according to the followers' intention to be recognized and to emerge.

## 5. Conclusion

In short, our study offers a contribution to the literature responding to the need expressed by González-Romá and colleagues [33] to study different variables as climate strength indicators. In doing so, this study follows the direction of some other studies that used consensus on transformational leadership [43, 66] and proposes an advancement by suggesting that the role of consensus on LMX be studied. When followers perceive that they are being treated in the same way, this could lead to different outcomes: They could perceive organizational justice in an environment where they perceive a resource loss (e.g., abusive supervision), but it may not be so effective when followers are experiencing positive situations (high job satisfaction) and are searching for motivational factors, striving to be recognized for their performance in order to emerge and stand out from the others.

## Figures and Tables

**Table 1:** Descriptive analyses and correlations table.

	*M*	SD	*ω*	r_WG(J)_	ICC_(1)_	ICC_(2)_	*D*_eff_	1	2	3	4
1. LMX consensus	0.94	0.04									
2. LMX	3.33	0.85	0.92	0.85	0.19	0.70	2.77	0.11^∗^			
3. Abusive supervision	1.43	0.07	0.92	0.91	0.09	0.52	1.87	−0.13^∗^	−0.57^∗^		
4. Job satisfaction	3.32	0.80	0.87	0.82	0.09	0.55	1.86	0.03	0.39^∗^	−0.26^∗^	
5. Internal turnover intention	2.17	1.32	0.91	0.33	0.11	0.61	2.09	−0.02	−0.28^∗^	0.31^∗^	−0.44^∗^

*Note*: *ω* = McDonald's *ω*; r_WG(J)_ = within-group interrater agreement index; ICC_(1)_/ICC_(2)_ = intraclass correlation coefficients; *D*_eff_ = design effect.

⁣^∗^*p* < 0.001.

**Table 2:** Unstandardized estimated coefficients from the tested multilevel models.

Dependent variable	Level 1 mediation		Cross-level moderations	
	Model 1 (*X* ⟶ *M* ⟶ *Y*)		Model 2	
	Job satisfaction	Internal turnover intentions	Job satisfaction	Internal turnover intentions
*Fixed effects*				
*Level 1*				
Intercept	3.76^∗∗∗^(0.08)	1.24^∗∗∗^(0.06)	3.32^∗∗∗^(0.03)	4.01^∗∗∗^(0.10)
Abusive supervision (*X*)	−0.30^∗∗∗^(0.05)	0.44^∗∗∗^(0.06)	−0.37⁣^∗∗∗^(0.04)	0.43⁣^∗∗∗^(0.07)
Job satisfaction (*M*)		−0.58^∗∗∗^(0.05)		−0.55^∗∗∗^(0.03)
Indirect effect (*X* ⟶ *Y*)		0.18^∗∗∗^(0.03)		
*Level 2*				
LMX consensus			0.47 ns (0.58)	−12.17^∗∗∗^(0.74)
*Interaction terms*				
LMX consensus ∗ abusive supervision			2.29^∗∗∗^(0.55)^a^	0.64 n.s. (1.12)^c^
LMX consensus ∗ job satisfaction				3.62^∗∗∗^(0.40)^b^
*Random effects*				
Level 1 variance	0.56^∗∗∗^(0.02)	1.23^∗∗∗^(0.06)	0.52^∗∗∗^(0.02)	1.20^∗∗∗^(0.07)
Level 2 variance	0.03^∗∗^(0.01)	0.13^∗∗∗^(0.04)	0.06^∗∗∗^(0.01)	0.14^∗∗∗^(0.03)

*Additional information*				
AIC	7218.660		7189.056	
BIC	7265.410		7282.555	
−2 ∗ log likelihood	7200.660		7153.056	
Parameters estimated	9		18	

*Note:* Standard errors are shown in brackets. *Y* = internal turnover intentions.

Abbreviations: AIC = Akaike Information Criterion, BIC = Bayesian Information Criterion.

^a^Moderation path a.

^b^Moderation path b.

^c^Moderation path c'.

⁣^∗^*p* < 0.05.

⁣^∗∗^*p* < 0.01.

⁣^∗∗∗^*p* < 0.001.

## Data Availability

The data that support the findings of this study are available from the corresponding author upon reasonable request.

## Endnotes

^1^FNOPI (2022): https://www.fnopi.it/wp-content/uploads/2022/10/AGENAS-personale_ssn_2022.pdf.

^2^Nurse24.it (2024): https://www.nurse24.it/infermiere/attualita-infermieri/crisi-infermieristica-italia-56-degli-infermieri-abbandona-ssn.html.

^3^NSI Nursing Solutions Inc. (2023): https://www.nsinursingsolutions.com/documents/library/nsi_national_health_care_retention_report.pdf.

^4^NursingUp (2023). https://nursingup.it/comunicati/500-de-palma-gli-infermieri-italiani-verso-un-punto-di-non-ritorno-sempre-piu-professionisti-scelgono-la-strada-delle-dimissioni-dalla-sanita-pubblica-diventata-come-una-insostenibile-prigione-di-vetro.html.

^5^FNOPI (2024): https://www.fnopi.it/2024/03/08/8-marzo-infermiere-3/

^6^Nurse24 (2022): https://www.nurse24.it/infermiere/attualita-infermieri/infermieri-italiani-sempre-piu-anziani.html.

## Supporting Information

The Strengthening the Reporting of Observational Studies in Epidemiology (STROBE) for the cross-sectional study checklist was submitted as supporting information to this study.
