# Supplementary material for: Effects of Consensus on Leader–Member Exchange (LMX) Within Nursing Teams on the Relationship Between Abusive Supervision, Job Satisfaction, and Unit Turnover: A Multilevel Moderation Study
Source: J Nurs Manag. 2025 Apr 1;2025:6220416. doi: 10.1155/jonm/6220416 (PMC11978477; doi:10.1155/jonm/6220416)
Supplement: Supporting Information — Additional supporting information can be found online in the Supporting Information section. [file 6220416.f1.doc]

STROBE Statement—Checklist of items that should be included in reports of ***cross-sectional studies***

|  | Item No | Recommendation | Page | Relevant text from manuscript |
| --- | --- | --- | --- | --- |
| **Title and abstract** | 1 | (*a*) Indicate the study’s design with a commonly used term in the title or the abstract | 1 | Effects of Consensus on Leader-Member Exchange (LMX) within Nursing Teams on the Relationship between Abusive Supervision, Job Satisfaction, and Unit Turnover: A Multilevel Moderation |
|  |  | (*b*) Provide in the abstract an informative and balanced summary of what was done and what was found | 1 | Aim. This study explores how consensus on leader-member exchange (LMX) — the degree of within-unit agreement regarding the LMX nurse leaders establish with each team member — moderates the effects of abusive supervision on job satisfaction and internal turnover intentions.  Method. Involving a sample of 1357 nurses nested into 130 groups (led by as many nurse leaders), cross-level moderations were tested.  Results. Results show that, on one hand, LMX consensus acts as a resource when it is stronger, dampening the effect of abusive supervision on job satisfaction. On the other hand, nurses with higher job satisfaction belonging to groups with higher LMX consensus report higher intentions to change wards than nurses in groups with lower LMX consensus. The discussion addresses the concept of “star employees”, i.e., employees with better performance, visibility, and relevant social capital.  Implications for Nursing Management. The discussion highlights the importance for nurse managers to consider both the quality of individual LMX and overall team consensus to enhance nurse well-being and reduce turnover intentions.  Keywords: consensus; LMX; multilevel moderation; climate strength; abusive supervision; job satisfaction; turnover intentions. |
| Introduction | | |  |  |
| Background/rationale | 2 | Explain the scientific background and rationale for the investigation being reported | 2-5 | “While the beneficial effects of positive leadership behaviors in enhancing nurses’ attitudes and behaviors, along with patient satisfaction and quality of care, are well-known [e.g., 1], healthcare organizations and leaders continue to face issues related to nurse well-being and turnover. In particular, since nurse leaders play a key role in influencing nurses’ well-being [2], it is vital for nurses’ perceptions about the behaviors of their leaders to be addressed, with a view to protecting their well-being. This is also a key feature in which to invest to improve nurse retention [3], as abusive supervision is one of the main antecedents prompting nurses to leave [4].”  “The act of quitting the organization is the culmination of a process in which “the work group can be considered as the first (work)place environment where employees develop cognition of leaving” [6, p. 755]. Since organizations are made up of a series of work groups, it would be restrictive not to consider the influence of group-level dynamics, mainly within a sample of nurses who develop strong relationships with their colleagues and supervisors on a daily basis [6]. Thus, exploring the moderating effects of LMX, conceived as group-level leadership climate strength [11], could both extend the climate strength concept to other constructs [12] and add new nuances to our understanding of individual-level leadership dynamics influenced by group-level consensus [13].” |
| Objectives | 3 | State specific objectives, including any prespecified hypotheses | 9-15 | “Thus, having explored the negative effects of abusive supervision on job satisfaction, its increasing effects on turnover intentions, and the mediating role of job satisfaction between leadership constructs and nurses’ intentions to leave the organization, we ultimately hypothesize that:  *H1: Abusive supervision has an indirect positive effect on internal turnover intentions via job satisfaction at within level.”*  *“Leadership consensus* is defined as “the shared perceptions of employees toward their direct supervisor” [13, p. 104].”  “Therefore, while studying within-unit consensus, the focus is placed on followers, managed by the same leader, assessing the level of consensus (or lack thereof) on the quality of the relationship established with their leader, which could be high or low. There could, for example, be a high consensus on a low-quality LMX; in this case, followers share the same perceptions about their leader. On the other hand, when followers do not agree (low consensus, high variability), they may feel they are being treated in different ways, and this could affect different psychological and organizational outcomes [11].”  “Given the impact of within-group agreement and unit turnover, it is interesting to shift the research focus towards exploring the effects of within-unit consensus in leadership dynamics on individual well-being outcomes and individual intentions to move between units in the same organization. For this reason, we hypothesize that:  *H2a: LMX consensus at the unit-level moderates the individual-level relationships between abusive supervision and job satisfaction, such that abusive supervision has a weaker negative effect on job satisfaction within teams characterized by high (versus low) LMX consensus.*  *H2b: LMX consensus at the unit-level moderates the individual-level relationships between job satisfaction and internal turnover intentions, such that job satisfaction has a stronger negative effect on internal turnover intentions within teams characterized by high (versus low) LMX consensus.*  *H2c: LMX consensus at the unit-level moderates the individual-level relationships between abusive supervision and internal turnover intentions, such that abusive supervision has a weaker positive effect on internal turnover intentions within teams characterized by high (versus low) LMX consensus.*” |
| Methods | | |  |  |
| Study design | 4 | Present key elements of study design early in the paper | 15-16 | “A sample of 1357 Italian nurses and 130 nurse leaders working in hospitals in northwestern Italy was involved, by filling out paper-and-pencil questionnaires.” […] “To match and merge the data of nurse leaders with their respective followers’ groups and at the same time respect the confidentiality of participants’ personal data, researchers generated alphanumeric codes. Participants were informed about the process through invitation letters and information sheets accompanying each questionnaire. Data collection started after the project obtained approval from the director of the Directorate of Health Professions and the nurse leaders of the target organization, allowing the participation of both leaders and their followers.” […] “Nurse leaders were invited via email, accompanied by an information sheet introducing the research characteristics. Upon agreement to participate, two administrators delivered paper copies of the questionnaires in person, collecting them soon after the completion. Nurses filled out their questionnaires, signed them with an alphanumeric code, and placed them in blank envelopes. The administrators collected all the envelopes from each ward.”  “The whole population of nurse leaders and nurses of the organization target was involved, counting 164 nurse leaders and 2664 nurses. Inclusion criteria in this study’s sample were nurse leaders consenting to complete the questionnaires, a minimum of three nurses per group participating in the questionnaire, and ensuring that at least 61% of all items were completed comprehensively. According to these criteria and after cleaning the data matrix from missing data, the final sample counts 1357 nurses nested into 130 groups (led by as many nurse leaders).” |
| Setting | 5 | Describe the setting, locations, and relevant dates, including periods of recruitment, exposure, follow-up, and data collection |  |  |
| Participants | 6 | (*a*) Give the eligibility criteria, and the sources and methods of selection of participants |  |  |
| Variables | 7 | Clearly define all outcomes, exposures, predictors, potential confounders, and effect modifiers. Give diagnostic criteria, if applicable | 18-19 | *“Job satisfaction* was measured with a 5-item scale, 4 items from the Copenhagen Psychosocial Questionnaire (COPSOQ) [48]. Items were adapted to the Italian language and used in previously published studies [20]. An example item is “How satisfied are you with the way your abilities are used?”, and one ad hoc item asks “How satisfied are you with your work as a whole, taking into consideration each element?”. Respondents were asked to answer using a 5-point Likert response scale, from 1 (“Very dissatisfied”) to 5 (“Very satisfied”). McDonald’s ω = .87.  *Internal turnover intention* was measured by adapting the formulation of the 3-item Intention to quit and turnover scale by Colarelli [49] with statements regarding changing the ward within the same organization. Participants were asked to answer using a 5-point Likert scale from 1 (“Strongly disagree”) to 5 (“Strongly agree”). An example item is “If I have my own way, I will be working for another unit/ward within this organization a year from now”. McDonald’s ω = .91.” |
| Data sources/ measurement | 8* | For each variable of interest, give sources of data and details of methods of assessment (measurement). Describe comparability of assessment methods if there is more than one group | 18-19 | *“Abusive supervision* was measured using the Italian validated version of Tepper’s [14] 15-item scale [47], with a 5-point Likert scale, ranging from 1 (“I cannot remember him/her ever using this behavior with me”) to 5 (“He/she uses this behavior very often with me”). A sample item is “My nurse leader gives me the silent treatment”. McDonald’s ω = .92.”  *“LMX* was assessed with the 7-item scale by Graen and Uhl-Bien [8], with a 5-point Likert response scale, which statement changes according to the item. The items were adapted to Italian. An example item is “Do you usually know how satisfied your leader is with what you do?”. McDonald’s ω = .92.”  “Then, in order to measure *LMX consensus* for every ward, the mean Euclidean distance was used, calculating the index with the following formula: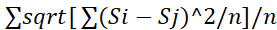. With this index, we averaged the dyadic differences between each individual participant (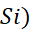 and the other members within the same working group (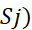, then we aggregated all the scores for dissimilarities for each ward ranged from 0 to 0.99, and finally, reversing the score we show that higher scores mean greater consensus. This approach is grounded in Chan’s [32] dispersion model, which conceptualizes consensus as a team-level configurational property [50]. The use of Euclidean distance specifically allows us to capture the variability in members’ perceptions by measuring the separation between individuals’ views and those of their group members.” |
| Bias | 9 | Describe any efforts to address potential sources of bias | 24 | “Prior to testing our hypothesized model, confirmatory factor analyses (CFA) were performed in order to ensure the independence of the variables and to check common method bias through Harman’s single factor method [62, 63]. A model in which variables load into only one factor shows not-so-good fit indices (ꭓ^2^_(435)_ = 14125.22, p < .001; CFI = .49; TLI = .45; RMSEA = .11 [.111; .115]; SRMR = .13), while the model considering four different dimensions shows better fit (ꭓ^2^_(435)_ = 14125.22, *p* < .001; CFI = .90; TLI = .89; RMSEA = .05 [.048; .053]; SRMR = .21), except for SRMR values. Therefore, considering the multilevel nature of our model, we performed a multilevel CFA, where only LMX consensus is analyzed at between level. Multilevel CFA shows a better fit (ꭓ^2^_(253)_ = 7680.60, *p* < .001; CFI = .92; TLI = .91; RMSEA = .05; SRMR_whithn_ = .05; SRMR_between_ = .00).” |
| Study size | 10 | Explain how the study size was arrived at | 21 | “The whole population of nurse leaders and nurses of the organization target was involved, counting 164 nurse leaders and 2664 nurses. Inclusion criteria in this study’s sample were nurse leaders consenting to complete the questionnaires, a minimum of three nurses per group participating in the questionnaire, and ensuring that at least 61% of all items were completed comprehensively. According to these criteria and after cleaning the data matrix from missing data, the final sample counts 1357 nurses nested into 130 groups (led by as many nurse leaders).”  “As regards the representativeness of the sample, its characteristics align with the general profile of nurses in Italy. Specifically, the gender distribution reflects the predominance of women in the nursing profession, both at the national (76.4%) and the specific regional levels (84.4%) ([link](https://www.fnopi.it/2024/03/08/8-marzo-infermiere-3/)). Similarly, the age distribution corresponds to the national trend of an aging nursing workforce, with a significant proportion being in their 40s or older (mean = 56.5 years old) ([link](https://www.nurse24.it/infermiere/attualita-infermieri/infermieri-italiani-sempre-piu-anziani.html)).” |
| Quantitative variables | 11 | Explain how quantitative variables were handled in the analyses. If applicable, describe which groupings were chosen and why | 19 | “<Leadership is inherently multilevel in nature> [53, p. 4], as individuals within a work group led by the same leader are <nested> within that leader. In multilevel studies, Level 1 (Individual Level) refers to individual perceptions and outcomes, while Level 2 (Group Level) comprises aggregated group-level variables, derived from individual responses within the same work unit. Leadership, as a social construct, operates across individual and group contexts. Leaders interact with individuals (Level 1) while simultaneously influencing the broader team environment (Level 2), and the multilevel model enables partitioning of variance between levels, ensuring robust estimates.”  “To test the multilevel moderated mediation model, with the moderator (i.e., LMX consensus) at the between level and all the variables implied in the mediation model, firstly, we group mean-centered the Level 1 predictor (i.e., abusive supervision) and grand mean-centered the Level 2 predictor (i.e., LMX consensus), then we used the “two-level random” type of analysis in Mplus, specifying “algorithm=integration” [54, 55].” |
| Statistical methods | 12 | (*a*) Describe all statistical methods, including those used to control for confounding | 19 | “To test the multilevel moderated mediation model, with the moderator (i.e., LMX consensus) at the between level and all the variables implied in the mediation model, firstly, we group mean-centered the Level 1 predictor (i.e., abusive supervision) and grand mean-centered the Level 2 predictor (i.e., LMX consensus), then we used the “two-level random” type of analysis in Mplus, specifying “algorithm=integration” [54, 55]. The two-level random approach in multilevel moderated mediation allows the random slope at Level 1 to be considered a latent variable at Level 2, used as an outcome variable to test the interaction effect [56]. This approach aligns with recommendations from the psychometric literature on analyzing multilevel models where both intercepts and slopes are considered outcome variables in cross-level moderation model [57]. To test the moderated mediation hypotheses of abusive supervision on internal turnover intentions via job satisfaction, with different conditions of LMX consensus moderating the different paths (a, b, and c’), i.e., high and low LMX consensus, we used the bootstrap estimates with the “integration=montecarlo” input to construct bias-corrected confidence interval (CI).  In order to justify aggregation and the multilevel analyses, r_WG(J)_, ICC_(1)_, ICC_(2)_ and D_eff_ were calculated [53, 58]. r_WG(J)_ is the index of within-group interrater agreement which assesses “agreement among the judgments made by a single group of judges on a single variable in regard to a single target” [59, p.5; 60]; ICC_(1)_ expresses the portion of the total variance of the variables that significantly vary across teams, i.e., localized in the upper level; besides it, ICC_(2)_ indicates the reliability of the scores at the higher-level analysis; finally, the design effect (D_eff_) measures how much the observations deviate from a hypothetical simple random sampling [61]. We adopted the following cut-off values: r_WG(J)_ between .51 and .70 shows a moderated agreement, while values greater than .70 show consistent agreement [58]; for ICC_(1)_ values greater than .05 are considered acceptable, while for ICC_(2)_ they should be greater than .70 [53]; finally, values of D_eff_ greater than 2 suggest the non-negligibility of clustering effects on the variables [61]. Our study showed sufficient values to proceed to multilevel analysis. Abusive supervision showed an r_WG(J)_ of .91; the ICC_(1)_ was .08 and the ICC_(2)_ was .52. For job satisfaction, r_WG(J)_ was .82, the ICC_(1)_ equal to .08 and the ICC_(2)_ equal to .55. Then, internal turnover intention showed an r_WG(J)_ of .33, an ICC_(1)_ of .11 and an ICC_(2)_ of .61. Lastly, LMX showed an r_WG(J)_ equal to .85, the ICC_(1)_ was .19 and the ICC_(2)_ equal to .70.” |
|  |  | (*b*) Describe any methods used to examine subgroups and interactions |  | / |
|  |  | (*c*) Explain how missing data were addressed |  | / |
|  |  | (*d*) If applicable, describe analytical methods taking account of sampling strategy |  | / |
|  |  | (*e*) Describe any sensitivity analyses |  | / |
| Results | | |  |  |
| Participants | 13* | (a) Report numbers of individuals at each stage of study—eg numbers potentially eligible, examined for eligibility, confirmed eligible, included in the study, completing follow-up, and analysed | 21 | “According to these criteria and after cleaning the data matrix from missing data, the final sample counts 1357 nurses nested into 130 groups (led by as many nurse leaders).” |
|  |  | (b) Give reasons for non-participation at each stage |  | / |
|  |  | (c) Consider use of a flow diagram |  | / |
| Descriptive data | 14* | (a) Give characteristics of study participants (eg demographic, clinical, social) and information on exposures and potential confounders | 21 | “The sample of nurses is comprised of 82.2% women and 17.8% men; the age range has a minimum of 22 and a maximum of 66 years (mean = 43.52, SD = 8.98); 53.6% have a Nursing school diploma, the 41.9% a Bachelor’s degree, and a 4.6% a Master’s degree. Nurses in the sample work within a university hospital network consisting of four large hospitals. Specifically, 51.6% of respondents are employed in the largest hospital district, and the remaining 20.5% in the trauma center hospital, 16.7% in the pediatric hospital, and 7.4% in the obstetrics and gynecology hospital. Additionally, 3.8% work in other hospitals located near the urban area. Moreover, 64.3% work in general medicine clinical area, while 28.4% in surgery, 14.4% in emergency room, and 19.9% in pediatrics. As regards specific wards, 22.9% work in general and specialized surgery, 21.4% in oncology, 11.9% in gynecology and obstetrics, 11.5% in pediatrics, 10.7% in orthopedics and rehabilitation, 9.2% in neurosciences, 5.8% in general and specialized medicine, 2.9% in emergency and intensive care, and 3.7% in other wards. Nurses have been working in the organization for an average of 17.57 years (SD = 9.74), while they have been working for their specific ward for a minimum of 1 year to a maximum of 41 years (mean = 10.71, SD = 8.48). Briefly, nurse leaders are composed of 84.6% women and 15.4% men, with an average age of 53.1 years (SD = 5.4). Among them, 58.5% work in the largest hospital district, 16.9% in the trauma center hospital, 16.2% in the pediatric hospital, 6.2% in the obstetrics and gynecology hospital, and 2.3% in other hospitals near the urban area. On average, nurse leaders have been coordinating their ward for 13.1 years (SD = 8.4) and have a total work experience of 32.6 years (SD = 6.27).” |
|  |  | (b) Indicate number of participants with missing data for each variable of interest |  | / |
| Outcome data | 15* | Report numbers of outcome events or summary measures | 26 | “Results show that at the individual level, abusive supervision negatively affects job satisfaction and it is positively related to internal turnover intentions; in turn, job satisfaction is negatively related to turnover intentions. Finally, the indirect effect of abusive supervision on internal turnover intentions via job satisfaction at within level is positive and significant (unstandardized estimate = .18; p < .001). Thus hypothesis 1, at the individual level, is confirmed. Then, adding the between-level LMX consensus index as moderator of the three paths (a, b, and c’), results show that LMX consensus significantly moderates the relationship between abusive supervision and job satisfaction (path a, confirming hypothesis 2a), and the one between job satisfaction and internal turnover intentions (path b, partially confirming hypothesis 2b, since the moderation is significant but in the opposite direction than expected), while the moderation of the direct relationship between abusive supervision and internal turnover intentions is not significant (path c’, disconfirming hypothesis 2c).” |
| Main results | 16 | (*a*) Give unadjusted estimates and, if applicable, confounder-adjusted estimates and their precision (eg, 95% confidence interval). Make clear which confounders were adjusted for and why they were included | 26-28 | “Finally, the indirect effect of abusive supervision on internal turnover intentions via job satisfaction at within level is positive and significant (unstandardized estimate = .18; p < .001).”  “Furthermore, the indirect effect of abusive supervision on internal turnover intentions via job satisfaction, with LMX consensus moderating path a, was calculated using the standard Montecarlo replications to build 95% CIs. Results show that it is stronger for groups with low (–1 SD) LMX consensus (unstandardized estimate = .27; 95% CI [.20; .27]) than for groups with high (+1 SD) LMX consensus (unstandardized estimate = .15; 95% CI [.09; .15]).”  “Furthermore, the indirect effect of abusive supervision on internal turnover intentions via job satisfaction, with LMX consensus moderating path b, was calculated by bootstrapping 10000 Montecarlo replications to build 95% CIs. Results show that it is stronger for groups with low (–1 SD) LMX consensus (unstandardized estimate = .20; 95% CI [.08; .31]) than for groups with high (+1 SD) LMX consensus (unstandardized estimate = .15; 95% CI [.05; .26]).” |
|  |  | (*b*) Report category boundaries when continuous variables were categorized |  | / |
|  |  | (*c*) If relevant, consider translating estimates of relative risk into absolute risk for a meaningful time period |  | / |
| Other analyses | 17 | Report other analyses done—eg analyses of subgroups and interactions, and sensitivity analyses |  | / |
| Discussion | | |  |  |
| Key results | 18 | Summarise key results with reference to study objectives | 28-29 | “In line with our hypotheses, consensus acts as a resource when it is stronger, dampening the effect of abusive supervision on job satisfaction.”  “However, and more interestingly, LMX consensus has some unexpected effects in moderating the relationship between job satisfaction and internal turnover intentions. Our results show that nurses belonging to groups with a higher level of LMX consensus (i.e., where there are similar perceptions among team members about the quality of the relationship with the same leader) report higher intentions to change wards than those of nurses belonging to teams with less similar perceptions when job satisfaction is high. We expected consensus to have been a resource for nurses, increasing their willingness to remain within a group with high consensus, where there is agreement within the team. However, this unexpected result suggests a more interesting theoretical explanation for this attitude among nurses.  Lastly, our results show that consensus is not effective in moderating the relationship between abusive supervision and internal turnover intentions, suggesting that when negative leadership behaviors are implemented by the leader, it is largely irrelevant whether nurses within the same group are treated in the same way or differently, as they choose another group and another leader.” |
| Limitations | 19 | Discuss limitations of the study, taking into account sources of potential bias or imprecision. Discuss both direction and magnitude of any potential bias | 35-36 | “Firstly, the use of self-reporting measures raises a concern about common-method bias. For this reason, we provided the results of the Harman’s single factor test in the result paragraph. Secondly, this research employs a cross-sectional design, limiting the ability to draw conclusions about the causal relationships between the variables under investigation. Thirdly, the model lacks an evaluation of the effect of certain control variables at both levels, which may act as confounding factors. We tested the correlation between the study’s key variables and several control variables, such as gender, age, tenure in the specific ward, tenure in the hospital, hours worked, affiliation with hospital macro-areas (Level 1), as well as the number of nurses in the team (Level 2). The Level 1 control variables either showed no correlation with the key study variables or only weak correlations (e.g., age and tenure in the hospital), without significant effects in the moderation model. Similarly, the Level 2 control variable had no significant effect in the moderation model. Therefore, we opted for a more parsimonious model, excluding the effects of control variables. Fourth, the generalizability of our findings may be influenced by cultural differences in leadership perceptions and behaviors, as highlighted by cross-cultural research, such as the GLOBE project [787]; thus, future studies could replicate our research in different cultural and healthcare settings to explore potential variations in the observed effects of the moderating variable. Lastly, it was not possible for us to distinguish followers while highlighting star employees according to some performance indices.” |
| Interpretation | 20 | Give a cautious overall interpretation of results considering objectives, limitations, multiplicity of analyses, results from similar studies, and other relevant evidence | 32-33 | “Thus, our research suggests different effects of consensus, considering relationships of different psychological dimensions and organizational outcomes. On the one hand, consensus about LMX seems to be a resource when faced with an abusive leader, capable of protecting nurses’ job satisfaction; on the other hand, when experiencing high levels of satisfaction, the fact that everyone is treated the same does not appear to be a motivational lever for remaining within the same work unit for nurses. Indeed, driven by the motivation to stand out [68] and to be recognized [69], nurses may tend to search for another work unit, which is often the first step towards leaving the organization [6]. These results are important since turnover has been confirmed to be one of the major issues for healthcare organizations due to the COVID-19 pandemic [74]. Indeed, studies conducted on the consequences brought about by the pandemic have demonstrated that lack of recognition by leaders was one of the main reasons linked to turnover intentions [75], suggesting both a deterioration of the problem and a greater need for leaders to pay attention to individual consideration and recognition of individual performances in order to strengthen nurses’ retention.” |
| Generalisability | 21 | Discuss the generalisability (external validity) of the study results | 35-36 | “Fourth, the generalizability of our findings may be influenced by cultural differences in leadership perceptions and behaviors, as highlighted by cross-cultural research, such as the GLOBE project [78]; thus, future studies could replicate our research in different cultural and healthcare settings to explore potential variations in the observed effects of the moderating variable. Lastly, it was not possible for us to distinguish followers while highlighting star employees according to some performance indices.” |
| Other information | | |  |  |
| Funding | 22 | Give the source of funding and the role of the funders for the present study and, if applicable, for the original study on which the present article is based | 15 | “This study is part of a broader project entitled “Feeling like a leader” whose focus was the study of leadership relationship features between nurse leaders and nurses of their working groups.”  Funder: Fondazione CRT.  Fondazione CRT funded principal investigator (i.e., the fourth author of this study) for the realization of the *“Feeling like a leader”* research project. |

*Give information separately for exposed and unexposed groups.

**Note:** An Explanation and Elaboration article discusses each checklist item and gives methodological background and published examples of transparent reporting. The STROBE checklist is best used in conjunction with this article (freely available on the Web sites of PLoS Medicine at http://www.plosmedicine.org/, Annals of Internal Medicine at http://www.annals.org/, and Epidemiology at http://www.epidem.com/). Information on the STROBE Initiative is available at www.strobe-statement.org.
